# The Bone Marrow Immune Microenvironment in CML: Treatment Responses, Treatment-Free Remission, and Therapeutic Vulnerabilities

**DOI:** 10.1007/s11899-023-00688-6

**Published:** 2023-02-13

**Authors:** Shaun David Patterson, Mhairi Copland

**Affiliations:** School of Cancer Sciences, College of Medical, Veterinary and Life Sciences, Paul O’Gorman Leukaemia Research Centre, University of Glasgow, 21 Shelley Road, Glasgow, G12 0ZD UK

**Keywords:** Chronic myeloid leukaemia, Leukaemia stem cells, Bone marrow immune microenvironment, Treatment-free remission, NK cells, Tyrosine kinase inhibitor

## Abstract

**Purpose of Review:**

Tyrosine kinase inhibitors (TKIs) are very successful for the treatment of chronic myeloid leukaemia (CML) but are not curative in most patients due to persistence of TKI-resistant leukaemia stem cells (LSCs). The bone marrow immune microenvironment (BME) provides protection to the LSC through multidimensional interactions, driving therapy resistance, and highlighting the need to circumvent these protective niches therapeutically. This review updates the evidence for interactions between CML cells and the immune microenvironment with a view to identifying targetable therapeutic vulnerabilities and describes what is known about the role of immune regulation in treatment-free remission (TFR).

**Recent Findings:**

Intracellular signalling downstream of the chemotactic CXCL12-CXCR4 axis, responsible for disrupted homing in CML, has been elucidated in LSCs, highlighting novel therapeutic opportunities. In addition, LSCs expressing CXCL12-cleaving surface protein CD26 were highly correlated with CML burden, building on existing evidence. Newer findings implicate the adhesion molecule CD44 in TKI resistance, while JAK/STAT-mediated resistance to TKIs may occur downstream of extrinsic signalling in the BME. Exosomal BME-LSC cross-communication has also been explored. Finally, further detail on the phenotypes of natural killer (NK) cells putatively involved in maintaining successful TFR has been published, and NK-based immunotherapies are discussed.

**Summary:**

Recent studies highlight and build on our understanding of the BME in CML persistence and TKI resistance, pinpointing therapeutically vulnerable interactions. Repurposing existing drugs and/or the development of novel inhibitors targeting these relationships may help to overcome these issues in TKI-resistant CML and be used as adjuvant therapy for sustained TFR.

## Introduction

Differentiated blood cells of all lineages are derived from rare and multipotent hematopoietic stem cells (HSCs) which exist in a quiescent state. HSCs differentiate ‘asymmetrically’ by simultaneously self-renewing and producing a distinct, more differentiated progeny cell. Progressive differentiation of hematopoietic stem and progenitor cells (HSPCs) to more lineage-committed progeny is accompanied by a loss of self-renewal in a tightly-regulated continuum, which eventually leads to a complement of differentiated blood cells. This topic is reviewed elsewhere [1].

The bone marrow (BM) is permeated by endothelium-lined vascular tissue which enables trafficking of mature blood progeny via permeable sinusoids [2]. The surrounding BM immune microenvironment (BME) is sparsely populated with mesenchymal stem/stromal cells (MSCs) which are characterised by immunophenotype, secretion profile, and localisation. Perivascular MSCs, periarteriolar MSCs, and perisinusoidal MSCs can be defined by surface Nestin expression (Nes^+^), neuron/glia antigen 2 (NG2), or leptin receptor (LepR) expression, respectively [3]. MSCs are heterogeneous and multipotent [4], giving rise to ‘niche’ BMEs which can support hematopoietic cells distinctly. For example, the arteriolar niche (containing NG2^+^ pericytes) is a more favourable environment for long-term quiescent HSCs than the sinusoidal niche (containing LepR^+^ stromal cells), which facilitates differentiation [5]. MSCs can also modulate extrinsic immune cell responses, such as aiding suppressive regulatory T cell (Treg) generation while suppressing effector CD4^+^ T cells [6].

**Fig. 1:**
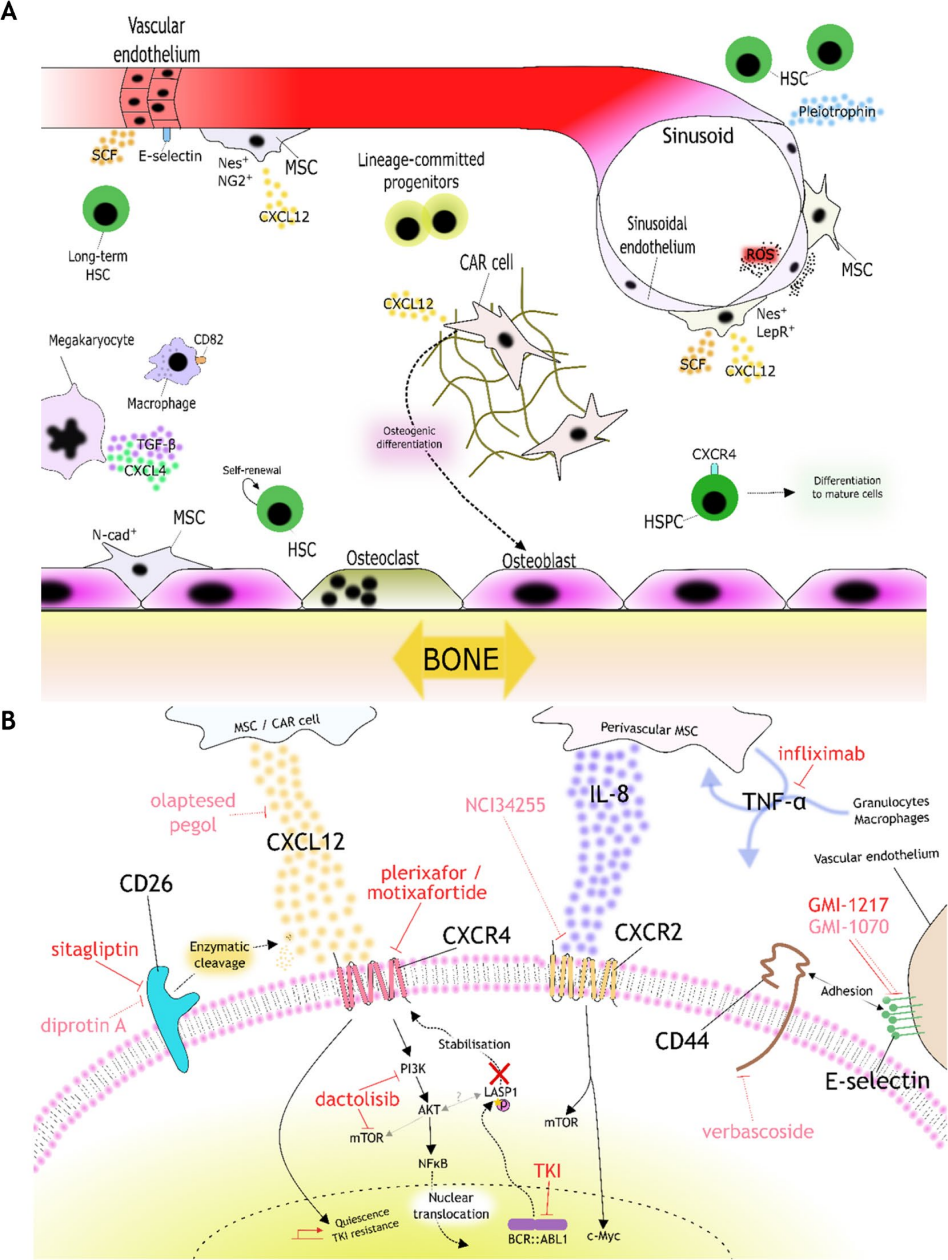
Schematic diagrams of the BME and targetable LSC-BME interactions in CML. **A** Selected features and interactions of the normal BME as described in the literature. **B** Selected interactions between the LSC and BME as described in the literature, with therapeutic targeting opportunities highlighted in red. Inhibitors in clinical investigation for CML or haematological malignancies shown by solid line; inhibitors in preclinical or non-hematological clinical investigation shown by dotted line. CAR, CXCL12-abundant reticular; HSC, hematopoietic stem cell; HSPC, hematopoietic stem and progenitor cell; LepR, leptin receptor; MSC, mesenchymal stem/stromal cell; N-cad, N-cadherin; Nes, nestin; ROS, reactive oxygen species; SCF, stem cell factor; TKI, tyrosine kinase inhibitor

Numerous soluble factors maintain and regulate hematopoiesis within the BME. The chemokine CXCL12 binds to HSCs via surface receptor CXCR4, and this relationship is key to HSC maintenance [7, 8]. Three-dimensional imaging has also identified widespread distribution of CXCL12-abundant reticular (CAR) cells throughout the BM [9•]. HSC function can be modulated by sinusoidal endothelium-derived pleiotrophin [10] and vascular endothelium-derived E-selectin [11]. In addition, there are other cellular regulators of hematopoiesis in the BME, including macrophages (via CD82) [12] and megakaryocytes (via CXCL4 and TGF-β signalling) [13, 14]. Finally, reactive oxygen species (ROS) at the sinusoidal niche help to drive HSPCs towards differentiation and trafficking into the circulation [2]. HSCs may also interact with the bone-oriented endosteal niche [15]. A schematic representation of selected BM niches is shown in Fig. 1A, providing the context for exploring the BME in chronic myeloid leukaemia (CML) leukemogenesis.

CML is a clonal malignancy which arises due to chromosomal translocation and the fusion of the *BCR* and *ABL1* genes. The resulting BCR::ABL1 fusion protein drives aberrant oncogenic signalling, expansion of malignant clones and clinical sequelae [16]. CML is usually diagnosed in the chronic phase (CP-CML), which can be managed long-term using BCR::ABL1-antagonising tyrosine kinase inhibitors (TKIs). A proportion of CP-CML may develop resistance to therapy or progress to blast phase (BP-CML), characterised by an acute condition, higher circulating blasts, and poor survival [17].

CML blasts are derived from a *BCR::ABL1*^+^ cell of origin termed a ‘leukaemia stem cell’ (LSC) due to its quiescence and self-renewal capacity [18, 19], akin to an HSC. LSCs are known to be resistant to TKIs through BCR::ABL1-independent mechanisms [20], and so can persist after successful TKI-induced remission, acting as a reservoir for CML relapse. Despite an improved understanding of LSC biology, they remain elusive and hard to target.

The mainstay of treatment for CP-CML involves BCR::ABL1-specific TKIs, and patients may be treated with multiple TKIs sequentially in order to attain/maintain a response. This review aims to update on the knowledge around the CML LSC, leukemic progeny, and the BME, in the context of therapeutic targeting, resistance, and treatment-free remission (TFR).

## CXCL12-CXCR4 Axis

The CXCL12-CXCR4 axis can sustain LSCs in addition to HSCs. In a CML mouse engraftment model, *Cxcl12* deletion in MSCs and CAR cells led to increased LSC cycling and TKI sensitivity, suggesting that stromal-derived Cxcl12 drives quiescence and TKI resistance. However, both undifferentiated LSC and progeny numbers increased, indicating that LSC self-renewal capacity was independent of Cxcl12 [21]. An earlier study demonstrated the formation of dense MSC/leukaemia progenitor clusters within the BM was also Cxcl12-driven [22], indicating that leukemic cells home towards a Cxcl12-rich niche.

Overexpression of the CXCL12 receptor CXCR4 in CML cells confers enhanced proliferative capacity and nilotinib resistance in vitro [23]. Chemotherapy resistance in K562 CML cells has also been associated with CXCR4 upregulation in addition to PI3K/AKT pathway activation and NF-κB nuclear translocation, highlighting an important signalling axis [24]. AKT phosphorylation downstream of CXCR4 activity has been described in K562 cells elsewhere [25].

In addition, the BCR::ABL1 target LASP1 was identified as an interactor of CXCR4 [23], in which low LASP1 activity/expression may contribute to the CXCR4-driven survival advantage of CML cells [26]. Butt et al. postulated that constitutive LASP1 phosphorylation by active BCR::ABL1 prevents CXCR4 stabilisation by LASP1, limiting CXCL12-CXCR4 signalling, and favouring cellular trafficking of CML cells from the BM into the periphery [27]. It has also been suggested that LASP1 is an intermediate player in CXCR4-AKT signalling [27].

Plerixafor is the only CXCR4 inhibitor in clinical use for autologous stem cell transplant (SCT), for which it aids peripheral mobilisation of CD34^+^ cells [28]. Plerixafor has not been evaluated clinically in CML. In relapsed acute myeloid leukaemia (AML), a phase I trial using Plerixafor demonstrated efficacy in overcoming stromal-leukaemia protection against targeted therapy, by mobilising CD34^+^CD38^−^ leukaemia cells to the periphery [29].

A similar strategy has been explored in CML using other agents. In vivo inhibition of CXCL12 in CML using a targeted RNA oligonucleotide (NOX-A12/olaptesed pegol) was synergistic with TKI, suggesting that enhanced CML mobilisation out of the BME sensitised these cells to BCR::ABL1 inhibition [30]. NOX-A12 has since shown an acceptable clinical safety profile in other cancers [31] and may be suitable for clinical development in CML.

## CD26

Disruption of the CXCL12-CXCR4 BM homing mechanism in leukemic cells has been partially attributed to activity of the surface enzyme dipeptidylpeptidase-IV (DDPIV/CD26) on LSCs. CD26 is expressed on CML LSCs as identified by the ability of BCR::ABL1^+^CD34^+^CD38^−^CD26^+^ LSCs to repopulate immunocompromised mice with CML, compared with CD26^−^ cells. The engraftment of CD26^+^ LSC was more diffuse in the BM which has been attributed to CD26-mediated CXCL12 cleavage and disruption to chemotactic behaviour [32]. The difference in engraftment of short-term- (ST-) and long-term (LT)-HSCs has been partially attributed to CD26 activity [33].

Identification of peripheral blood (PB) CD34^+^CD38^−^CD26^+^ cells was found to be diagnostic for CML in patients with a suspicious leukocytosis [34], supporting previous evidence and highlighting CD26 as a CML LSC biomarker. This has been confirmed by a prospective clinical study in which CD26 stratified suspected haematological malignancies into CML and non-CML [35].

CD34^+^CD38^−^CD26^+^ PB cells were correlated with *BCR::ABL1* transcript in early CP-CML [36]. In contrast, interim analyses of the PROSPECTIVE FLOWERS study revealed that PB *BCR::ABL1*^+^ measurement was not associated with CD26^+^ LSC levels after initiation of TKI therapy, although CD26^+^ LSC levels at diagnosis could predict subsequent responses to TKIs [37]. The correlation of CD26^+^ LSC numbers in the PB and BM [38] may reflect their disrupted BM-homing ability, but also support its utility as a predictive biomarker.

Gliptins antagonise CD26 enzymatic activity and are clinically used in resistant diabetes mellitus, due to the catalytic action of CD26 on incretins. Gliptins can also impair CD26-mediated stromal interactions in vitro [32]. Pre-exposure of CD26^+^ LSC to vildagliptin was limited their engraftment in mice [32], although another study found no effect of vildagliptin on CML engraftment (± imatinib) when mice were treated in vivo [39]. However, this latter model selected only CD34^+^ cells and there may have been sub-optimal targeting of CD26 LSCs at this vildagliptin dose in vivo. There may also be inter-species differences between murine and human stroma.

In addition to gliptins, CD26 enzymatic activity was effectively blocked in HSCs by the small molecule inhibitor diprotin A in in vivo models of the hematopoietic system [33], but is not utilised clinically. Additionally, use of CD26-targeting immunoliposomes loaded with the BCL-2 inhibitor venetoclax showed promising anti-LSC effects in vitro [40], and may worthy of further exploration in vivo.

## CXCR2 Signalling and TNF-α

An in vivo study demonstrated the upregulation of Cxcl1 on murine stromal cells (akin to human perivascular MSCs) in CML, with upregulation of LSC Cxcr2, the Cxcl1 receptor. This interaction maintained self-renewal in LSCs, but not HSCs, and was mediated by TNF-α signalling [41]. In humans, IL-8/CXCL8 binds to CXCR2 and is the equivalent to murine Cxcl1. The differential dependence of HSCs and LSCs on IL-8 signalling may provide a therapeutic window in CML. In AML, the blockade of IL-8 binding to CXCR2 using a novel experimental inhibitor (NCI34255) led to a reversal of therapy resistance [42], highlighting a means of therapeutically targeting IL-8/CXCR2 signalling in CML.

Monoclonal antibody (mAb) targeting of TNF-α with infliximab enhanced the effect of TKIs against LSCs, although IFN-γ-modulating effects of infliximab may be contributing to the observed effects [43]. Alternatively, CXCR2 signalling in CML may function via mTOR and c-Myc [44], identifying other therapeutic possibilities.

## Adhesion Mechanisms

Evidence suggests that LSC quiescence is induced/maintained by LSC CD44 binding to vascular E-selectin, increasing therapy resistance. Godavarthy et al. elucidated that CD44 is negatively regulated transcriptionally by *SCL/TAL1* within *BCR::ABL1*^+^ LSCs and that imatinib led to CD44 upregulation by *SCL/TAL1* [45]. Baykal-Kӧse et al. demonstrated that CML cells upregulate CD44 to adapt phenotypically to TKI-induced stress [46]. Using a CML murine engraftment model, combined E-selectin inhibitor GMI-1217 (uproleselan) and imatinib led to improved survival and reduced BM endothelial adherence, compared to imatinib alone, supporting the hypothesis that E-selectin mediated adhesion by CML LSCs (via CD44) is an imatinib resistance mechanism [45]. A phase I/II trial of GMI-1217 in relapsed/refractory AML (NCT02306291) demonstrated an acceptable safety profile and promising efficacy, in combination with chemotherapy [47]. A phase III trial of GMI-1217 in a similar context is ongoing (NCT03616470) [48], which may support the clinical development of GMI-1217 for CML. E-selectin antagonist GMI-1070 was clinically safe in a phase III trial for sickle cell disease [49] but has not been trialled in hematologic malignancies. Other in vitro evidence suggests that CD44 mediates resistance to imatinib downstream of *AF1q* — a putative BCR::ABL1-independent oncogene [50].

Plant-derived verbascoside can block CD44 dimerisation [51], and it was shown to induce apoptosis in a model of CML, possibly by mediating p38-MAPK/JNK and Caspase-3 signalling, although the role of CD44 was not explored here [52]. CD44 is highly expressed within normal, CML and AML CD34^+^CD38^+^ and CD34^+^CD38^−^ BM cells [53•], likely making it unsuitable as a CML LSC biomarker and a cancer-specific therapeutic target, but downstream signalling may be targetable.

Previous reports suggested CML LSCs were more dependent on L-selectin (CD62L) for BM homing and disease engraftment [54]. Soluble CD62L is elevated in CP-CML patients and reduced following imatinib treatment [55], suggesting it may have value as a marker of treatment response. In addition, soluble and T cell expression of CD62L may predict responses to TKIs in CML [56], indicating this marker may be more related to T cell responses than the CML cells themselves. Plasma levels of soluble adhesion molecule VCAM-1 (CD106) may also reflect responses to TKI [57].

## IL1RAP and CD36

Landberg et al. have described the expression of LSC markers IL1RAP and CD36 which can separate *BCR::ABL1*^+^ from *BCR::ABL1*^−^ populations and predict responses to TKI [58, 59]. IL1RAP is, as part of the IL-1 receptor (IL-1R) complex, responsive to cytokines such as IL-1β and IL-33 and may function via NFκB, as shown in K562 cells [60]. It is also expressed on BM MSCs. In vivo data have shown that chimeric antigen receptor T (CAR-T) cells targeting IL1RAP may be a viable option to eliminate MRD/LSCs and ‘cure’ CML [61].

IL1RAP^+^ LSCs can be separated into two subsets by CD36 expression [59]. CD36 is a fatty acid transporter which, in HSCs, has been shown to permit entry to the cell cycle in situations of emergency haematopoiesis (e.g., during infection) [62]. Previous evidence has suggested that CD36^+^ CML LSCs mediate chemoresistance via increased fatty acid oxidation, and home to adipose tissue niches within the BM [63]. Other preliminary data suggests that CD36 may not be essential for healthy hematopoietic cells [64], highlighting another potential therapeutic window.

## CD93

Kinstrie et al. identified a novel population of primitive Lin^−^CD34^+^CD38^−^CD90^+^ LSCs expressing the transmembrane receptor CD93 which persisted in CML patients despite extended TKI treatment [65••]. Elsewhere, CD93 expression was found on CML LSCs, co-expressed with such markers as CD25 and IL1RAP, but also on some HSC populations [53•]. However, Riether et al. demonstrated that although both LSCs and normal HSCs expressed CD93, it was only required for self-renewal capacity in LSCs. In addition, the clinically-approved anti-sickness drug metoclopramide was found to inhibit CD93 and downstream signalling, therefore blocking LSC activity in vivo [66•]. This has not yet been evaluated clinically in CML.

## Exosomal Crosstalk

Exosomes are cell-derived lipid nanoparticles containing genomic material which are involved in cell–cell communication. *BCR::ABL1*-containing exosomes can be detected in CP-CML patient plasma [67] and their release from CML cells can stimulate pro-survival signalling from BM MSCs [68]. CML cells can remodel the BME to facilitate a leukaemia-favourable niche using exosomes. For example, micro-RNA (miRNA) exosomal transfer to BM MSCs by CML cells was shown to inhibit the normal osteogenic function of these MSCs [69]. In the reverse direction, MSC-derived exosomes can promote CML cell survival and TKI resistance, putatively through cell extrinsic means (e.g., enhanced angiogenesis around the niche) [70]. Exosomal membrane proteins linked to TKI resistance, such as CD36, have been identified in CML and may represent candidate biomarkers for further development [71].

Exosomal communication can also impact on other immune populations within the BME. Ex vivo exposure of cord blood T cells to CML-derived exosomes led to upregulation of the exhaustion marker PD-1, which can be bound by its ligand PD-L1 to inhibit T cell activation. In addition, skewing towards a Treg phenotype was observed in this model [72], suggesting exosome-induced alteration of T cells favours immunosuppressive phenotypes which are permissive of CML leukemogenesis. Similarly, CML-derived exosomes can increase levels of anti-inflammatory IL-10 and immune cell-blocking ROS in the BME, in addition to skewing macrophages away from anti-tumour phenotypes [73].

## T Cell Exhaustion

The concept of T cell exhaustion refers to the impairment of T cell effector function which often arises during malignancy due to chronic antigen exposure, although definitions vary [74]. T cell expression of exhaustion markers such as PD-1 [75], TIM-3, and LAG-3 [76] have been demonstrated to reflect the leukaemia burden in patients, and decrease during effective responses to TKI therapy. In addition, immunosuppressive Tregs have been shown to reflect leukaemia burden [76]. As such, reversal of leukaemia-induced T cell exhaustion may be a therapeutic opportunity. Inhibition of TIM-3 ± PD-1 or LAG-3 has been described as effective against solid tumours and there is particular interest into applying these in AML [77]. There is an ongoing phase III trial in chronic myelomonocytic leukaemia (CMML) — a rare disease which is related, but distinct, to CML — investigating the efficacy of anti-TIM-3 antibody sabstolimab on overall survival.

## Modulating PD-1/PD-L1 Signalling

PD-1/PD-L1 signalling is a known mechanism of T cell suppression. PD-1 (CD279) is typically expressed by T and B cells, while its ligand PD-L1 is expressed on a wide number of cell types, and often on tumour cells as a means of immune escape [78].

PD-L1-carrying exosomes derived from BM MSCs transfer to tumour cells of varying phenotype in vivo [79], demonstrating a mechanism by which MSCs can encourage immune escape by oncogenic cells. CML cells may also influence innate immune populations such that they express higher levels of PD-L1 [80], potentially as a mechanism of T-cell anti-tumour suppression.

Inhibition of the PD-1/PD-L1 axis is established as an anti-leukemic strategy. There is an ongoing trial investigating the safety and efficacy of combined anti-PD-1 antibody pembrolizumab with TKIs in CP-CML [81•]. Other means of inhibition may counteract PD-L1 activity. BRD4 degradation was found to reduce PD-L1 upregulation on CML LSCs, which is attributed to both inhibition of IFN-γ-mediated upregulation and because the *PDL1* gene is a target of BRD4 [82]. Pharmacological inhibition of inflammatory mediators IRAK1 and IRAK4 may also reduce PD-L1 expression on CML LSCs [83].

## Hypoxia and PI3K

Chronic hypoxia within the BME has been well-studied in the context of AML LSC pathogenesis. CML cells are sensitive to hypoxia [84], and the critical regulator of hypoxia responses, hypoxia-inducible factor 1α (HIF1α), is essential for CML LSC survival in the BME [85]. Recent evidence demonstrated that BP-CML cells displayed an altered balance of available ROS and antioxidant species in the cell — or redox balance — due to a perturbed synergism between HIF1α and opposing Notch1 [86]. Metabolic rewiring of LSCs in response to the unique BME conditions, such as chronic hypoxia, modulates the cells’ responses to therapy and offers myriad of therapeutic targeting opportunities; this topic is covered elsewhere [87].

Inhibition of the PI3 kinase (PI3K) pathway may be effective against the LSC-BME interactome, particularly the hypoxic context. Oxidative stress induced by hypoxia was shown to be mitigated by adaptive PI3K signalling in ex vivo murine BM-MSCs, downstream of leukaemia initiating factor (LIF) [88], highlighting niche-targeting opportunities.

Simultaneous inhibition of PI3K (± mTOR) signalling with copanlisib (or BEZ235, respectively) was shown to effectively block osteoblast proliferation and prevent osteoblast-mediated resistance to TKIs in CML LSCs [89]. While this suggests that PI3K is important to LSC-extrinsic means of TKI resistance, other evidence has shown that CML cell intrinsic BCR::ABL1 signalling via the PI3K/AKT/mTOR axis also drives TKI resistance/cell survival, via other players including epidermal growth factor receptor kinase substrate 8 (EPS8) [90] and c-Myc [91]. A clinical trial of copanlisib in acute relapsed/refractory leukaemias (but not CML) was well tolerated at lower doses [92].

## Inhibition of Janus Kinases

JAK/STAT signalling is an intracellular regulator of hematopoietic cell function [93] and has been explored as a therapeutic target within TKI-resistant CML. The JAK1/2 inhibitor ruxolitinib is clinically approved for myeloproliferative neoplasms and was shown to effectively impair repopulation of immunodeficient mice by self-renewing CML HSPCs, attributed to JAK/STAT5 inhibition primarily [94]. JAK2 inhibition with ruxolitinib could also impair CML cell immune evasion by preventing the BCR::ABL1-independent MHC-II downregulation that has been observed in CML HSPCs [95]. Zhang et al. demonstrated in vivo that BM-MSC-derived Il-7 was a mechanism of BCR::ABL1-independent JAK1/STAT5-mediated TKI resistance in CML cell lines [96]. These data underpin the rationale for combined TKI and JAK inhibition to overcome therapy resistance.

Combination therapy of ruxolitinib and nilotinib was explored in a recent phase I trial which reported promising efficacy of the combination [97], although another ruxolitinib/TKI combination trial in CML (NCT01751425) was stopped early due to lack of efficacy (data not published). Trials targeting the BME in CML are summarised in Table 1, and a schematic representation of targeting opportunities in the BME is shown in Fig. 1B.

**Table 1:** Interventional clinical trials investigating BME- or immune-modulating therapies for CML or in CML-relevant contexts. Information on trials was extracted from the clinicaltrials.gov database, and trial registration numbers are reported in the table

Desired target/pathway	Inhibitor	Type of inhibitor/mechanism	Relevant clinical trial(s)	Relevant clinical trial information
CXCL12/CXCR4	Plerixafor	CXCR4 antagonist; Small molecule	Non-CML malignancies Healthy volunteers	Stem cell mobilisation
	Olaptesed pegol	CXCL12-targeting; RNA aptamer	Healthy volunteers	NCT00976378 (completed) First-in-human phase I study: for autologous SCT
	Motixafortide	CXCR4 antagonist; Synthetic peptide	CML	NCT02115672 (withdrawn) Phase I/II: CP-CML patients not responding to imatinib
				NCT02639559 (ongoing) Phase II: CD34^+^ cell mobilisation for SCT to CML patients
CD26	Sitagliptin	CD26 antagonist; small molecule	CML, AML, ALL, Lymphoma, MDS	NCT01720264 (ongoing) Phase II: enhance umbilical cord blood (UCB) transplantation engraftment
JAK/STAT	Ruxolitinib	JAK1/2 antagonist; small molecule	CP-CML	NCT01702064 (completed) [97] Phase I: ruxolitinib and nilotinib combination
				NCT03654768 (ongoing) Phase II: ruxolitinib and TKI
				NCT03610971 (ongoing) Phase II: ruxolitinib and TKI to enhance second TFR
E-selectin	GMI-1217/uproleselan	E-selectin antagonist	Relapsed/refractory AML	NCT03616470; phase III (ongoing) [48] NCT02306291; phase I/II (completed) [47]
TNF-α	Infliximab	Chimeric anti-TNF-α antibody	MDS	NCT00074074 (completed) Phase II
PI3K	Dactolisib	PI3K/mTOR; small molecule	BP-CML, AML, ALL	NCT01756118 (unknown) Phase I: relapsed/refractory acute leukaemia
TIM-3	Sabatolimab	Humanised anti-TIM-3 antibody	CMML, MDS	NCT04266301 (ongoing) [100] Phase III: sabatolimab combination with azacitidine
PD-1	Pembrolizumab	Humanised anti-PD-1 antibody	CP-CML	NCT03516279 (ongoing) Phase II: pembrolizumab/TKI combination
NK cells	KDS-1001	NK cells as immunotherapy	CP-CML	NCT04808115 (ongoing) Phase I: NK cell therapy to eliminate MRD in CML

## Other Secreted Factors

Other secreted factors within the BME have more recently been highlighted in CML pathogenesis. Himburg et al. found that pleiotrophin, which is normally produced by BM-MSCs, is upregulated in CML HSPCs downstream of *BCR::ABL1* induction in vivo and leads to cell self-maintenance, independently of MSCs [98]. Successful inhibitors of pleiotrophin have not been described in recent years. Separately, a cytokine screen of CML patient samples identified myostatin propeptide (MSTNpp) to significantly stimulate CD34^+^CD38^low^ proliferation in vitro, independently of its known ligand myostatin. In addition, MSTNpp plasma levels were found to be the same in CML and non-CML patients, suggesting unsuitability as a biomarker, although it could be investigated as a therapeutic target. This study also identified soluble CD14 (Scd14), IL-21, IL-13 variant (IL-13v), and CCL28 as important for CML CD34^+^CD38^low^ expansion [99] and should be investigated further. A summary of putative and established biomarkers for CML are summarised in Table 2.

**Table 2:** Putative CML biomarkers. Putative biomarkers in CML (as discussed in the text) are described with the appropriate references shown in the final column. The site of tissue from which each marker was studied is given as described in the reference, in addition to relevant information derived from these studies

Biomarker	Site	Category/putative function	Additional notes	Reference(s)
CD26^+^ LSC	PB	Diagnostic for CML LSC		[34, 35]
		Measurement of *BCR::ABL1*^+^ disease burden	PB and BM levels of CD26^+^ LSC are equivalent	[36, 38]
		Predictor of response to TKI at diagnosis		[37]
IL1RAP^+^ LSC	BM	Predictor of response to TKI at diagnosis		[58]
CD36^+^ LSC	BM	Marker of response to TKI	Assessed within first 3 months of TKI	[59]
CD93^+^ LSC	BM	Putative predictor of resistance to TKI		[65••]
T-cell PD-1 expression	PB	Marker of leukaemia burden / response to TKI		[75, 101]
T-cell TIM-1 and/or LAG-3 expression	PB	Marker/putative predictor of refractory disease	Predictive ability of biomarker was not assessed in this study specifically	[76]
Exosomal proteins	PB plasma	Marker of imatinib resistance		[102]
miRNAs	PB	Marker/putative predictor of response to TKI	miRNAs described hsa-miR-181a-5p hsa-miR-182-5p hsa-miR-26a-5p	[103]
sCD62L	PB plasma	Marker/putative predictor of response to TKI	T-cell surface CD62L also interrelated with sCD62L and can predict responses	[55, 56]

## Treatment-Free Remission

CP-CML patients on TKIs who achieve prolonged deep molecular remission (DMR; *BCR::ABL1* transcript ≤ 0.01% on the International Scale [IS]) can attempt to stop TKIs permanently without relapse, termed ‘treatment-free remission’ (TFR). Long-term TFR rates of up to 70% have been reported clinically [104]. However, only a minority of patients achieve DMR and hence can attempt TFR. Hypotheses as to why only some patients sustain TFR often focus on the anti-leukemic role of the host immune system, particularly within the BME.

Some evidence has focused on natural killer (NK) cell populations in TFR. A recent study found that TFR could be predicted by expression of NK receptors NKG2A and NKG2D, which enable stronger priming of NK cells for cytotoxic responses [105]. This is in agreement with another recent study [106] but contrasts others, which either found no differences in NKG2A/NKG2D expression comparing TFR vs. relapsing patients [107] or that elevated NK cells were associated with relapse [108]. Single-cell analyses have shown NK cells in TFR patients display a more activated phenotype than healthy controls, suggesting the importance of cytotoxic NK responses in controlling *BCR::ABL1*^+^ residual cells [109••].

Many of these studies assessed PB NK cells which may not capture any differential phenotype of BME-derived NK cells. In CML patient BM, but not PB, a terminally mature CD57^+^ subset was significantly higher than in healthy controls, indicating a possible role for BME influences on the differentiation status of these NK cells in CML specifically [110]. This is reflective of previous observations made in PB [75]; however, the importance of CD57.^+^ NK cells to TFR is unclear. The use of expanded NK cell immunotherapy to eliminate MRD in CML is being explored clinically [111] and may represent an option for adjuvant therapy in patients attempting TFR. Other novel NK-based therapeutics include anti-leukemic mAbs which induce targeted NK antibody-mediated responses and CAR-NK cell therapy, neither of which is described for CML, but for other malignancies, as reviewed by Allison et al. [112]

## IFN-α as an Adjuvant Therapy

Interferon alpha (IFN-α) was a mainstay of treatment for CP-CML prior to the introduction of TKIs into standard practice [113]. Combination therapy of IFN-α and TKIs has recently garnered interest as a means of achieving DMR more rapidly than TKIs alone (discussed by Talpaz et al. [114]). Some evidence suggests IFN-α could be combined with later generation TKIs for the effective treatment of BP-CML [115].

Limited evidence suggests IFN-α is an effective adjuvant for sustained TFR with short TKI duration, although one-third of patients did not tolerate IFN-α side effects in this study [116]. In another study, CML patients with DMR who took IFN-α were also more likely to stay in TFR at 18 months post-TKI cessation, although the rate of TFR in the non-IFN-α group was considerably lower than reported in other trials [117], suggesting some differences in the cohort/methodologies to other TFR trials.

Patients attempting TFR (in DMR) who had been treated with both IFN-α and TKIs prior to discontinuation showed a stronger memory-like phenotype in the NK cell compartment (defined by NKG2C^+^) and a Th1-skewed T cell response [118•]. Proportions of CD56^bright^ NK cells were reported to increase in patients attempting TFR who also received IFN-α therapy after TKI discontinuation, as opposed to those who did not [119]. It seems counterintuitive that CD56^bright^ NK cells, which are weakly cytolytic, are elevated in patients who sustain TFR, based on previous assumptions about the anti-tumour role of cytotoxic NK cells. Another study found that CML patients co-treated with IFN-α and TKI had higher PB populations of immune cells typically seen as ‘immunosuppressive’ including CD56^bright^ NK cells and granulocytic myeloid-derived suppressor cells (Gr-MDSCs) [120], which again conflicts with the idea that IFN-α enhances sustained TFR. Further characterisation of immune function in TFR is required to complement these studies.

## Conclusion

While TKIs continue to be effective for long-term management of CP-CML, the achievement of sustained DMR is a challenge for many patients. Additionally, a shift in focus to maintaining long-term TFR stresses the need to eliminate MRD and the *BCR::ABL1*^+^ LSC in CML. Newer evidence highlights the protective role of BM niches towards the LSC and the complex immune microenvironment(s) which facilitate TKI resistance, therefore identifying novel therapeutic vulnerabilities. Ongoing clinical investigation explores the feasibility of some approaches in CML and other haematological malignancies which manipulate the BME. Overall, bringing modern CML therapy beyond TKIs alone, increasing the number of patients that can attempt and successfully maintain TFR.

## Data Availability

There is no research data within this article for which availability is required.

## Abbreviations

BM: Bone marrow
BME: Bone marrow microenvironment
BP-CML: Blast phase chronic myeloid leukaemia
CAR: CXCL12-abundant reticular
CML: Chronic myeloid leukaemia
CMML: Chronic myelomonocytic leukaemia
CP-CML: Chronic phase chronic myeloid leukaemia
DMR: Deep molecular remission
DPPIV: Dipeptidylpeptidase-IV
Gr-MDSC: Granulocytic myeloid-derived suppressor cell
HIF1α: Hypoxia-inducible factor 1α
HSC: Hematopoietic stem cell
HSPC: Hematopoietic stem and progenitor cell
IL-1R: IL-1 receptor
ILK: Integrin-linked kinase
IS: International scale
LepR: Leptin receptor
LIF: Leukaemia initiating factor
LSC: Leukaemia stem cell
LT-HSC: Long-term hematopoietic stem cell
mAb: Monoclonal antibody
MSC: mesenchymal stem/stromal cell
MSTNpp: myostatin propeptide
N-cad: N-cadherin
Nes: nestin
NG2: neuron/glia antigen 2
NK: natural killer
PB: peripheral blood
PI3K: PI3 kinase
qRT-PCR: quantitative real-time PCR
ROS: reactive oxygen species
ST-HSC: short-term hematopoietic stem cell
TFR: treatment-free remission
TKI: tyrosine kinase inhibitor
Treg: regulatory T cell
